# Supplementary material for: Protocol for a cluster randomized study to compare the effectiveness of a self-report distress tool and a mental health referral service to usual case management on program completion among vulnerable youth enrolled in a vocational training program
Source: PLoS One. 2024 Aug 1;19(8):e0294806. doi: 10.1371/journal.pone.0294806 (PMC11293660; doi:10.1371/journal.pone.0294806)
Supplement: S1 Protocol — (DOCX) [file pone.0294806.s001.docx]

Protocol for a pragmatic, open-label, 2x2 factorial, cluster randomized, superiority study to compare the effectiveness of adding a remote self-reporting tool for distress and a fit-for-purpose mental health & addictions service to usual case management on program completion and employment among unemployed visible minorities and women enrolled in a publicly funded vocational construction trades training program

*TeachMeToBuild* Study

Protocol version: version 1.0 31-AUG-2022

This protocol is the confidential intellectual property of the Principal Investigator and Sponsor. Acceptance implies an agreement not to disclose information contained herein that is not otherwise publicly available and without the express permission of the Principal Investigators.

# Table of Contents

[Table of Contents 2](#_Toc113023584)

[1 Trial Registration 6](#_Toc113023585)

[1.1 Data set 6](#_Toc113023586)

[1.2 Glossary of abbreviations and terms 8](#_Toc113023587)

[2 Protocol version 8](#_Toc113023588)

[3 Funding 8](#_Toc113023589)

[4 Administrative information 8](#_Toc113023590)

[4.1 Investigators 8](#_Toc113023591)

[4.2 Community Builders 8](#_Toc113023592)

[4.21 Administrative 9](#_Toc113023593)

[4.3 Royal Victoria Regional Health Centre 9](#_Toc113023594)

[4.31 Administrative, Mental Health & Addictions 9](#_Toc113023595)

[4.4 Trial Sponsor 9](#_Toc113023596)

[4.41 Contact Information 9](#_Toc113023597)

[4.42 Administrative 9](#_Toc113023598)

[4.43 Roles and responsibilities 10](#_Toc113023599)

[4.43a *Trial Sponsor* 10](#_Toc113023600)

[4.43b *Trial funders* 10](#_Toc113023601)

[5 Background and Rationale 10](#_Toc113023602)

[5.1 Background 10](#_Toc113023603)

[5.11 Youth unemployment 10](#_Toc113023604)

[5.12 Vocational training programs 11](#_Toc113023605)

[5.13 Barriers to vocational program participation and completion 12](#_Toc113023606)

[5.14 Care management 12](#_Toc113023607)

[5.15 Trades & Diversity Training Program (TDTP) 13](#_Toc113023608)

[5.2 Rationale 14](#_Toc113023609)

[5.3 Choice of comparator 15](#_Toc113023610)

[6 Hypotheses 15](#_Toc113023611)

[6.1 Primary 15](#_Toc113023612)

[6.2 Secondary 16](#_Toc113023613)

[7 Objectives 16](#_Toc113023614)

[7.1 Primary 16](#_Toc113023615)

[7.2 Secondary 16](#_Toc113023616)

[8 Design 17](#_Toc113023617)

[9 Study Setting 17](#_Toc113023618)

[10 Eligibility 18](#_Toc113023619)

[10.1 Inclusion criteria 18](#_Toc113023620)

[10.2 Exclusion criteria 18](#_Toc113023621)

[11 Interventions 18](#_Toc113023622)

[11.1 Distress thermometer 18](#_Toc113023623)

[11.12 Web-based, self-report tool for distress 19](#_Toc113023624)

[11.2 Mental health & addictions 20](#_Toc113023625)

[11.3 Compliance 21](#_Toc113023626)

[12 Outcomes 21](#_Toc113023627)

[12.1 Primary outcome measures 21](#_Toc113023628)

[12.11 Program attendance 21](#_Toc113023629)

[12.12 Program completion 21](#_Toc113023630)

[12.13 Post-program employment 22](#_Toc113023631)

[12.2 Secondary outcome measures 22](#_Toc113023632)

[12.21 Mental Health & Addictions support 22](#_Toc113023633)

[12.22 Healthcare utilization 22](#_Toc113023634)

[12.23 Apprentice Satisfaction 22](#_Toc113023635)

[12.24 Case Management Utilization 23](#_Toc113023636)

[12.25 Acceptability of self-report tool 23](#_Toc113023637)

[12.26 Feasibility of self-report tool 23](#_Toc113023638)

[12.27 Compliance of self-report tool 24](#_Toc113023639)

[13 Participant timeline 24](#_Toc113023640)

[14 Sample size and power 25](#_Toc113023641)

[14.1 Difference in proportion of AFDs 25](#_Toc113023642)

[14.2 Difference in proportions of TDTP completion 26](#_Toc113023643)

[14.3 Difference in proportions of full-time employment 27](#_Toc113023644)

[15 Recruitment 27](#_Toc113023645)

[16 Allocation 28](#_Toc113023646)

[16.1 Sequence generation 28](#_Toc113023647)

[16.2 Concealment mechanism 28](#_Toc113023648)

[16.3 Implementation 29](#_Toc113023649)

[17 Masking 29](#_Toc113023650)

[18 Data Collection 29](#_Toc113023651)

[18.1 Methods 29](#_Toc113023652)

[18.11 Distress Thermometer data 29](#_Toc113023653)

[18.12 Routinely collected data 31](#_Toc113023654)

[18.13 Study-specific data 32](#_Toc113023655)

[18.14 Healthcare utilization 33](#_Toc113023656)

[18.2 Retention 33](#_Toc113023657)

[18.21 Strategies 33](#_Toc113023658)

[18.22 Withdrawal 34](#_Toc113023659)

[19 Data management 34](#_Toc113023660)

[20 Statistical methods 35](#_Toc113023661)

[20.1 Outcomes 35](#_Toc113023662)

[20.11 Primary 35](#_Toc113023663)

[20.12 Secondary 36](#_Toc113023664)

[21 Monitoring 38](#_Toc113023665)

[22 Harms 38](#_Toc113023666)

[23 Auditing 38](#_Toc113023667)

[23.1 Investigator responsibilities 38](#_Toc113023668)

[23.2 Coordinating centre responsibilities 38](#_Toc113023669)

[23.3 Site initiation 38](#_Toc113023670)

[23.4 Study monitoring 38](#_Toc113023671)

[23.5 Site close-out 39](#_Toc113023672)

[23.6 Source documents 39](#_Toc113023673)

[23.7 Direct access to data and documents 39](#_Toc113023674)

[24 Ethics approval 39](#_Toc113023675)

[25 Protocol amendments 40](#_Toc113023676)

[26 Consent 40](#_Toc113023677)

[27 Confidentiality 40](#_Toc113023678)

[28 Declaration of interests 41](#_Toc113023679)

[29 Access to data 41](#_Toc113023680)

[30 Post-trial care 41](#_Toc113023681)

[31 Trial results and authorship 41](#_Toc113023682)

[32 Reproducible research 42](#_Toc113023683)

[33 References 42](#_Toc113023684)

[34 Budget 45](#_Toc113023685)

[35 Appendix 47](#_Toc113023686)

[35.1 National Centre for Vocational Education Research Student Outcomes Survey 47](#_Toc113023687)

[35.2 Foundational skills assessment 48](#_Toc113023688)

[35.3 Distress Thermometer 49](#_Toc113023689)

[35.4 Expressed Consent Presentation 55](#_Toc113023690)

# 1 Trial Registration

## 1.1 Data set

| Data Category | Information |
| --- | --- |
| Primary registry and trial identifying number | ClinicalTrials.gov (NCT05626374) |
| Date of registration in primary registry | November 23, 2022 |
| Secondary identifying numbers | N/A |
| Source(s) of monetary or material support | Alectra (Royal Victoria Hospital Foundation) |
| Primary sponsor | Royal Victoria Regional Health Centre  Research Institute |
| Contact for public queries | Shawna Bailey [shawna@communitybuilders.co] |
| Contact for scientific queries | Giulio DiDiodato  [didiodatog@rvh.on.ca] |
| Public title | Comparing the effect of adding a remote self-reporting tool for distress and fit-for-purpose mental health & addictions service to usual case management on dropout rates in a vocational training program |
| Scientific title | A pragmatic, open-label, cluster randomized, 2x2 factorial, superiority study to compare the effectiveness of adding a remote self-reporting tool for distress and a fit-for-purpose mental health & addictions service to usual case management on program completion and employment among unemployed visible minorities and women enrolled in a publicly funded vocational construction trades training program |
| Country of recruitment | Canada |
| Problem studied | Dropout rates and post-program employment in vocational programs |
| Intervention | Experimental: Web-based, self-reporting tool and fit-for-purpose Mental Health & Addictions program  Active Comparator: Active case management |
| Key inclusion and exclusion criteria | Inclusion criteria:  1) Enrollment in Trades & Diversity Training Program  2) Written informed consent prior to enrolment  3) Must have an active Ontario Health Insurance Plan number  4) Must have a valid Canadian Social Insurance Number  5) Access to Wi-Fi  6) Must be between the ages of 18 and 49 years of age  Exclusion criteria:  1. Language barrier compromises the participant’s ability to complete the self-report tool for distress |
| Study type | Interventional  Allocation: 2x2 factorial, cluster randomization  Intervention model: parallel assignment  Masking: open-label  Primary purpose: comparative effectiveness |
| Date of first enrollment | TBD |
| Target sample size | 400 (fixed by program funding and requirements) |
| Recruitment status | Not yet started |
| Primary outcome(s) | 1) Difference in proportion of program completion  2) Difference in absence-free program days (time-frame: program completion 12 weeks)  3) Difference in proportion with full-time employment (time frame: 24 months post-program completion) |
| Secondary outcome(s) | 1) Difference in utilization of healthcare visits (time frame: during program (12 weeks) and 24 weeks post-program completion)  2) Difference in time to access mental health and addiction services  3) Difference in National Centre for Vocational Education Research Student Outcomes Survey Satisfaction scores  4) Acceptability of self-report tool  5) Feasibility of self-report tool  6) Compliance with self-report tool  7) Difference in mean hours of navigator case management |

## 1.2 Glossary of abbreviations and terms

AFDs Absence-free program days

BCM Brokered Case Management

ICF Informed Consent Form

ICM Intensive Case Management

MH&A Mental Health & Addictions

NEET Neither in employment, education or training

PHIPA Personal Health Information Protection Act

REDCap® Research Electronic Data Capture

ROC Receiver operating curve

RVRHC Royal Victoria Regional Health Centre

TDTP Trades & Diversity Training Program

# 2 Protocol version

| 2022-August-31 | Original (version 1.0) |
| --- | --- |
|  |  |

# 3 Funding

Alectra Fund for Health and Social Innovation

Royal Victoria Regional Health Centre Foundation

Employment & Social Development Canada (ESDC), *Skilled Trades Awareness & Readiness Program*

# 4 Administrative information

## 4.1 Investigators

Giulio DiDiodato MD, PhD (Principal Investigator)

Chief Research Scientist

Royal Victoria Regional Health Centre

Email: [didiodatog@rvh.on.ca](mailto:didiodatog@rvh.on.ca)

Telephone: (705)728-9090 extension 45641

Assistant Professor

Department of Health Research Methods, Evidence & Impact

McMaster University

1280 Main Street West, 2C, Hamilton, Ontario, Canada, L8S 4K1

[didiodatog@mcmaster.ca](mailto:didiodatog@mcmaster.ca)

## 4.2 Community Builders

## 4.21 Administrative

Shawna Bailey BA (Psychology)

Director of Programming, Communications, and Resource Development

Community Builders

Email: [shawna@communitybuilders.co](mailto:shawna@communitybuilders.co)

Telephone: (705)881-1076 extension 307

## 4.3 Royal Victoria Regional Health Centre

## 4.31 Administrative, Mental Health & Addictions

Carrie Stoner BA (Psychology), R.S.S.W.

Interim Manager – Integrated Crisis Services

Royal Victoria Regional Health Centre

Email – [stonerc@rvh.on.ca](mailto:sotnerc@rvh.on.ca)

Telephone (705)728-9090 extension 49627

Brian Irving RCS, RP, CACII, CMP

Manager, Addiction Services

North Simcoe Muskoka RAAM Services

Adult Mental Health Day Program & Community Treatment

Royal Victoria Regional Health Centre

Email: [irvingbr@rvh.on.ca](mailto:irvingbr@rvh.on.ca)

Telephone: (705)728-9090 extension 24311

## 4.4 Trial Sponsor

## 4.41 Contact Information

Royal Victoria Regional Health Centre (RVH) Research institute

Sponsor’s Reference: 119129260 RR 0001 (Charitable Registration Number)

Contact name: Jesse McLean PhD

Address: Health Library, Office 2218c, Royal Victoria Regional Health Centre, 201 Georgian Drive, Barrie, Ontario, L4M 6M2

Telephone: (705)728-9090 extension 41350

Email: [mcleanje@rvh.on.ca](mailto:mcleanje@rvh.on.ca)

## 4.42 Administrative

Jesse McLean PhD

Manager, RVH Research Institute

Royal Victoria Regional Health Centre

Health Library, Office 2218c, 201 Georgian Drive, Barrie, Ontario, L4M 6M2

Email: [mcleanje@rvh.on.ca](mailto:mcleanje@rvh.on.ca)

Telephone: (705)728-9090 extension 41350

Kelly Cruise BHSc, CCRP

Clinical Research Coordinator, RVH Research Institute

Royal Victoria Regional Health Centre

Research Office, 201 Georgian Drive, Barrie, Ontario, L4M 6M2

Email: [cruisek@rvh.on.ca](mailto:cruisek@rvh.on.ca)

Telephone: (705) 728-9090 extension 45639

## 4.43 Roles and responsibilities

## 4.43a *Trial Sponsor*

The trial sponsor has no role in the design, analyses, interpretation of the data, writing or decision to report the study results. The sponsor will support study coordination and execution, including informed consent, enrollment, randomization, follow-up, administering questionnaires, data collection and storage, and monitoring. The study sponsor is responsible for taking all reasonable steps to ensure proper conduct of the clinical trial protocol, and that the clinical trial is performed in accordance with the International Council for Harmonisation for Good Clinical Practice (<https://www.ich.org/page/efficacy-guidelines#6>) and all applicable regulatory requirements.

## 4.43b *Trial funders*

The funding sources have no role in the design, execution, analyses, interpretation of the data, writing or decision to report the study results.

# 5 Background and Rationale

## 5.1 Background

### 5.11 Youth unemployment

In 2019, 67.3% of young Canadians aged 15 to 30 were employed (1), a rate that was approximately 15% lower than older individuals. Employment rates are lowest among those aged 15 to 19 (60.2%), compared to rates exceeding 80% in those older than 20. Part of these differences are due to lower participation rates among young Canadians aged 15 to 19 due to school attendance. However for those not in school, these Canadians were also more likely to be unemployed (over 15%) compared to those aged 20 and older (<10%). Among young Canadians, there were minor differences in employment rates between men/women and Canadian-born/immigrants. In contrast, education levels had a profound impact on differences in employment rates. For those without a high school diploma, only 52.2% were employed compared to high school graduates (75%) or post-secondary education (>88%). Even after accounting for education, employment rates among Indigenous youth are 20% lower than their non-Indigenous counterparts. Of those employed, 44.3% of those aged 15 to 19 are employed in full-time permanent jobs, meaning they work ≥30 hours per week and have no pre-determined end date, while another 53.2% work in either part-time or non-permanent jobs, a rate that is 2-fold higher than those aged over 20. The rate of involuntary part-time or non-permanent employment has also displayed a disturbing trend with a rate that is almost 10% higher today compared to 30 years ago, reflecting increased difficulty in finding full-time employment. Some young Canadians are neither in employment, education nor training (NEET), and they are at high risk of chronic unemployment, social disengagement and poor quality of life. The prevalence of NEET among young Canadians is as high as 12.2% in men and 14.8% in women, with little differences due to education or immigrant status. Among Indigenous youth, approximately 3 out of 10 never finish high school, and unemployment rates can be as high as 70% on reserves (2). Visible minorities, persons other than Indigenous peoples who are non-Caucasian in race or non-white in colour, are also disadvantaged in employment opportunities (2,3). For example, even among university-educated visible minorities, the unemployment rate is 50% greater than non-racialized groups. Identifying these young Canadians and providing them with career skills training and opportunities is crucial for their full participation in society.

### 5.12 Vocational training programs

Vocational training programs provide students, sometimes referred to as apprentices, the educational opportunity to develop a skilled trade such as electrical or carpentry. These training programs alternate academic sessions and practicums, where practicums provide an opportunity to gain hands-on experience under the supervision of a mentor who is an experienced trades person. By the end of the training program, students will have acquired the skills required to perform the work. Many construction trades in Canada are recognized as Red Seal trades (4). The Red Seal designation is a nationally-recognized standard for skilled trades workers in Canada. Some of these Red Seal trades include bricklayer, cabinetmaker, carpenter, concrete finisher, construction craft worker, construction electrician, drywall finisher and plasterer, floorcovering installer, gasfitter, insulator, painter, plumber, refrigeration and air conditioning mechanic, roofer, pipefitter, and tile setter (5). The Red Seal designation is a nationally-recognized standard for skilled trades workers in Canada. The Red Seal designation establishes learning outcomes and objectives for vocational trades training programs that promote consistency across jurisdictions and prepare apprentices who have successfully completed their training to attempt the Red Seal Exam and receive a Red Seal endorsement and trade certification.

The impact of skilled trades in Canada is enormous, with approximately 1 in 5 Canadians employed in sectors of the Canadian economy employing skilled trades. Both men and women with skilled construction trades certification earn more than their counterparts with either a high school or college diploma (6). In addition, the long-term impact at 2 or more years after program completion demonstrates an average increase in employment rates of 5 to 12% (7). In 2020, the latest period for which we have data, there were 46 536 apprentices registered in vocational construction trades programs in Ontario, a decline of 4.5% from the previous year (8). Over 31% were under 24 years of age, but only 4.7% of those registered were females. Even fewer females, 60 in 2020, had achieved Red Seal certification in their construction trade, a decline of over 37% from the previous year. In the last 20 years, there has only been a single year, 2018, in which over 100 females, 117 in total, had achieved Red Seal certification in their skilled construction trade. These Red Seal-certified females in construction trades earned at least 2- to 3-fold more annually than females employed in other service trade industries (9), such as hairstylist and esthetician, food services, and early childhood educator and assistant programs that accounted for 59.1% of all females registered in apprenticeship programs in 2020 (10). However, these females continued to earn less than their similarly trained and certified male counterparts in the construction trades. For example, in 2015, a female construction electrician earned on average $56 310 (n=150) compared to $71 480 for males (n=5 670). A significant part of this disparity in income may be related to the gross underrepresentation of females in these male-dominated skilled trades (6). Overall, women in male-dominated apprenticeship programs, those where males account for over 75% of all registrations, are less likely to be employed after training, less likely to be employed in a job related to their trade, work fewer hours, and earn lower median wages (6).

### 5.13 Barriers to vocational program participation and completion

An effective vocational training program has been defined as “one which, within the confines of the existing policy and economic environment, is designed and delivered in a way that is well aligned with the learning needs of its target population such that it enables participation and produces relevant skill gains that lead to beneficial labour market outcomes.” (11). Unemployed individuals with low education and/or low life-skills, including low literacy skills, are the usual targets for vocational training programs. Additional barriers to successful completion of training and subsequent employment include mental health and substance use disorders, family responsibilities, disabilities, language barriers, transportation barriers and criminal records (5). These barriers need to be identified and addressed as part of any vocational training program in order to maximize the chances of successful program completion and subsequent employment. Programs that provide support services to mediate these barriers and enable apprentice participation and persistence are also more successful (11). Other program design features associated with success include small class sizes, a strong hands-on training component to establish connections with employer networks, and skill trades certification (11).

### 5.14 Care management

There is a growing recognition that support services should be provided to those apprentices most at risk of drop-out and unemployment (3,12). There are 2 recognized models of support services; wraparound and case management (12). Wraparound is described as an individual-specific, team-based approach to the identification, planning and coordination of support services. Case management is described as the development of a consistent and trusting relationship between a single point of contact and the individual at-risk in order to identify, plan and coordinate support services. Wraparound services focus on developing a unique care plan for each at-risk individual that is then implemented as the template guiding interactions between the care team and the individual. In general, wraparound support service teams provide comprehensive care management to each individual and there is only occasional need to seek out additional supports or services external to the team. As a result of the increased frequency and regularity of interactions, wraparound support service teams are restricted to small caseloads, typically less than 10 per team. While case managers may also regularly meet with at-risk individuals, there is no *a priori* care plan that guides these interactions, and case management is usually focused on reacting to active issues that emerge during training that may compromise the apprentice’s continued participation in the program. Most often, case managers must reach out to external services and supports to assist the at-risk individual. This is referred to as a Brokered Case Management (BCM) model (13,14). BCM is one type of a standard case management model. Intensive case management (ICM) models provide more comprehensive services beyond referral services that may involve a team that provides fit for purpose services.

### 5.15 Trades & Diversity Training Program (TDTP)

The TDTP is a federally-funded vocational construction skills training program. TDTP is funded through the Government of Canada’s Skilled Trades Awareness and Readiness Program initiative (15), which focuses on helping individuals who face barriers to employment to develop skills and experience they need to find a job or continue their education. The TDTP has been developed and will be delivered by Community Builders®, a social enterprise located in Minesing, Ontario, Canada (<https://www.communitybuilders.co/community-impact/training-and-employment/>). The TDTP is not part of a formal education program and does not lead to formal Red Seal certification for any of the construction trades. As part of the TDTP, Community Builders® provides a Case Manager for each cohort who regularly interacts with each apprentice to help them navigate external services and supports for any barriers that may exist or arise during training that threaten their ongoing participation or completion. TDTP is a cohort-based construction skills training program, with anticipated class sizes between 8 and 15 participants. Each cohort will experience structured activities over a 12-week period that include both in-classroom education and supervised hands-on experiential learning at participating construction sites. The in-class component will account for 22% (102 total hours) of the 12-week program. The in-class component will be led by the Construction Trainer, Case Manager, and Program Director. The in-class component will focus on teaching life skills needed for employment, referred to as the socio-economic & construction curriculum. The curriculum will include lessons about, but not be limited to the following: trade-specific vocabulary; trade math skills; ways to effective communication; conflict resolution in the workplace (referred to as soft skills); health and safety in the workplace; career / trade exploration; and interview and resumé-writing skills. The remaining 78% (360 total hours) will be spent on-site in supervised, hands-on learning. TDTP will target visible minorities, individuals who are Indigenous, and women for enrollment.

Throughout the TDTP, the Case Manager teaches soft skills each Monday morning for three hours in-person in a classroom type setting. This teaching involves slide decks on a specific topic, hand-outs, discussion, and reflection questions. The Program Director teaches for 60 minutes on topics which include trade math skills, career exploration, and health & safety in the workplace. The Construction Trainer is involved in direct hands-on teaching with the apprentices on a variety of topics included in residential construction (drywall, painting, framing, flooring, etc.). Teaching on Mondays are at a ratio of one teacher to 8 to 15 apprentices. From Tuesdays to Thursdays, apprentices are out on job-sites with Community Builders’ mentors (ratio being 1:1). During these periods, apprentices are involved in hands-on work skills training with all aspects of residential construction, while being encouraged to use the learned soft skills (for example, conflict resolution). During the 12-week program, apprentices and all Community Builders’ staff use the *Slack* platform (<https://slack.com/>) to communicate with each other. This communication can be private (1:1 messages) or in a group channel.

Problems with apprentices are typically identified quickly by any of the of the Community Builders’ staff. While apprentices are encouraged to reach out to the Case Manager if they are feeling any distress, different types of issues are also identified through 1:1 conversations that may happen on the work sites with their mentors. If a construction mentor identifies that there is an issue that the apprentice is experiencing, they will reach out through *Slack* or directly contact the Case Manager. Once this occurs, the Case Manager initiates a conversation with the apprentice to explore the distressful issue(s). Where appropriate, the Case Manager will coach and assist the apprentice in setting goals and working through the issue(s). In circumstances that require external consultation to help resolve, the Case Manager will refer the apprentice to appropriate external resources (for example, the Canadian Mental Health Association).

## 5.2 Rationale

In previous iterations of the TDTP, the Case Manager and Program Director have developed relationships with many other external agencies and service providers to facilitate access for their clients. Most of these relationships have involved basic communication and referrals, and would be described as minimally active relationships (16). For some problems, however, such as mental health and drug and alcohol addictions, the Case Manager has experienced difficulty in timely access to healthcare services. These delays have had negative consequences for program attendance and completion (Shawna Bailey, Program Director, personal communication). The Program Director has indicated that a more cooperative and coordinated relationship (16) with these services might benefit their clients. To this end, Community Builders® is partnering with Mental Health & Addictions (MH&A) services at the Royal Victoria Regional Health Centre (RVRHC) to develop a fit-for-purpose referral and access process that will ensure appropriate and timely care for those at-risk apprentices. As part of this novel case management strategy, we will also introduce a remote self-reporting tool for distress that will be completed by apprentices and monitored by the Case Manager to pre-emptively identify apprentices at-risk of absenteeism or drop-out with the hope that early identification and intervention will be more effective than the usual case management approach. A real-world (pragmatic) randomised study is therefore needed to compare the effectiveness of this Intensive Case Management approach compared to the usual Brokered Case Management approach for apprentices enrolled in the TDTP. This study is needed to demonstrate the superior benefits of ICM on important outcomes such as improved skills training retention and completion and post-training employment in order to justify its implementation across other vocational training programs with at-risk youth.

## 5.3 Choice of comparator

The study interventions will be allocated according to a 2x2 factorial design (Figure 1).

**Figure 1: 2x2 factorial design for case management strategies**

|  | Self-report tool for distress | |
| --- | --- | --- |
| MH&A Service | Control | Intervention |
| Control (Sudbury) | BCM | ICM^10^ |
| Intervention (Barrie) | ICM^01^ | ICM^11^ |

In this 2x2 factorial design, the intervention effects for 3 ICM models will be estimated. For the cohorts located in Sudbury, the current standard of case management will be the BCM model. The reason for this is the absence of a working relationship between Community Builders® and the MH&A program at Health Sciences North, the healthcare authority for the greater Sudbury region, that would permit the establishment of a similar fit for purpose service as the one created for the Barrie cohort. In this BCM model, the Training Program Coordinator in Sudbury will host bi-weekly meetings with each apprentice to ensure that all boundaries to skilled trades employment are eliminated. This includes, but is not limited to, check-ins (frequency) with the apprentice through Slack, frequent visits to the construction sites, and monitoring of the feedback forms that mentors complete weekly on the attendance, attitude, and coachability of their apprentice. For the ICM^10^ and ICM^01^ models, this BCM model will serve as the control for estimating the effects of the self-report for distress tool and the MH&A program, respectively. For the ICM^11^ model, both the ICM^10^ and the ICM^01^ models will serve as the controls to estimate the interaction effect for the combined self-report for distress tool and the MH&A program.

# 6 Hypotheses

## 6.1 Primary

In apprentices enrolled in the TDTP who are at-risk of absenteeism, drop-out or unemployment post-program completion, exposure to either the ICM^10^ or ICM^01^ models will be superior to the BCM model for reducing absenteeism (as determined by the number of absence-free days), drop-out (as determined by the proportion of apprentices completing the program), and unemployment (as determined by proportion employed at 24 months post-program completion).

## 6.2 Secondary

In apprentices enrolled in the TDTP who are at-risk of absenteeism, drop-out or unemployment post-program completion, exposure to the ICM^11^ model will be superior to either the ICM^10^ or ICM^01^ models for reducing absenteeism (as determined by the number of absence-free days), drop-out (as determined by the proportion of apprentices completing the program), and unemployment (as determined by proportion employed at 24 months post-program completion).

# 7 Objectives

## 7.1 Primary

1) To compare the effect of ICM^10^ and ICM^01^ versus BCM on reducing absenteeism in apprentices enrolled in TDTP

2) To compare the effect of ICM^10^ and ICM^01^ versus BCM on reducing drop-out rates in apprentices enrolled in TDTP

3) To compare the effect of ICM^10^ and ICM^01^ versus BCM on reducing unemployment rates in apprentices enrolled in TDTP

4) To compare the effect of ICM^11^ versus ICM^10^ and ICM^01^ on reducing absenteeism in apprentices enrolled in TDTP

5) To compare the effect of ICM^11^ versus ICM^10^ and ICM^01^ on reducing drop-out rates in apprentices enrolled in TDTP

6) To compare the effect of ICM^11^ versus ICM^10^ and ICM^01^ on reducing unemployment rates in apprentices enrolled in TDTP

## 7.2 Secondary

1) To compare the effect ICM^10^ and ICM^01^ versus BCM on time to access mental health & addiction services

2) To compare the effect ICM^10^ and ICM^01^ versus BCM on utilization of healthcare visits

3) To compare the effect ICM^10^ and ICM^01^ versus BCM on National Centre for Vocational Education Research Student Outcomes Survey Satisfaction scores

4) To compare the effect ICM^10^ and ICM^01^ versus BCM on mean hours spent on case management by the Case Manager

5) To compare the effect of ICM^11^ versus ICM^10^ and ICM^01^ on time to access mental health & addiction services

6) To compare the effect of ICM^11^ versus ICM^10^ and ICM^01^ on utilization of healthcare visits

7) To compare the effect of ICM^11^ versus ICM^10^ and ICM^01^ on National Centre for Vocational Education Research Student Outcomes Survey Satisfaction scores

8) To compare the effect of ICM^11^ versus ICM^10^ and ICM^01^ on mean hours spent on case management by the Case Manager

9) To measure acceptability of using self-report tool for distress by apprentices and Case Manager

10) To measure feasibility of using self-report tool for distress by apprentices and Case Manager

11) To measure compliance of using self-report tool for distress by apprentices

# 8 Design

The *TeachMeToBuild* trial is designed as a pragmatic, open-label, 2x2 factorial, cluster randomized, controlled, superiority study. Each cohort (cluster) will be randomized in a 1:1 allocation ratio to the self-report tool for distress using a stratified, permuted-block group schema according to the following strata:

1) Study site (Sudbury, Barrie)

The MH&A fit-for-purpose service will only be available to apprentices located at the Barrie site.

# 9 Study Setting

Community Builders® is a not-for-profit construction-based social enterprise (<https://www.communitybuilders.co/about-us/>) with offices in Simcoe County (Barrie) and Greater Sudbury. The enterprise offers training and employment to populations at risk of chronic unemployment such as NEET youth. The enterprise has had previous experience in apprenticeship training over the last 5 years, with 42 apprentices enrolled in their vocational training programs. The city of Barrie is located in Central Ontario with a population of approximately 147 000 (<https://www.city-data.com/canada/Barrie-City.html>). Approximately 20% of the population is between the ages of 15 to 30 years. The unemployment rate is 6.0%. 93% of residents speak English only, with 1% French only. Only 6.7% of citizens are visible minorities, and 2.1% identify as Indigenous. Prevalence of low income after taxes is 11.6%. Barrie is one of Canada’s fastest growing communities, with a growth rate exceeding 4.5% over the previous 5 years. The city of Sudbury is located in Northern Ontario with a population of approximately 85 000 (<https://www.phsd.ca/resources/research-statistics/health-statistics/2016-demographic-profile-public-health-sudbury-districts/>). Approximately 13% of the population is between the ages of 15 to 30 years. The unemployment rate is 8.5%. 70% of residents speak English only, with 24.7% French only. Only 1.2% of citizens are visible minorities, and 17.5% identify as Indigenous. Prevalence of low income after taxes is 11.6%. The population growth rate was less than 1% over the last 5 years. In each city, the TDTP will consist of both in-class and on-site learning opportunities as previously described.

# 10 Eligibility

Participants must provide written, informed consent before any study procedures occur.

## 10.1 Inclusion criteria

Participants eligible for the trial must comply with all of the following at randomization:

1. Enrolled in one of the TDTP cohorts

2. Must have an active Ontario Health Insurance Plan number

3. Must have a valid Canadian Social Insurance Number

4. Access to Wi-Fi network

5. Must be between the ages of 18 to 49 years of age

## 10.2 Exclusion criteria

1. Language barrier (non-English or French) compromises the participant’s ability to complete the self-report tool for distress

# 11 Interventions

## 11.1 Distress thermometer

This study will use the Distress Thermometer screening tool to create a self-reporting, web-based application to monitor distress in apprentices that may lead to absenteeism, drop-out or unemployment (17,18). The Distress Thermometer is a validated tool for identifying and measuring the severity of psychological distress. While originally validated in cancer patients, it has been used in many other clinical and non-clinical populations (19). The Distress Thermometer is a single-item visual analog scale from 0 (no distress) to 10 (extreme distress). The patient is asked to rate their level of distress over the previous week using this scale. The threshold for concerning levels of distress varies depending on the clinical context, but has been found to range between 3 and 5 in cancer patients, with the median cut-off of 4 maximising the sensitivity (median 0.83, range 0.5 to 1.0) and specificity (median 0.68, range 0.36 to 0.98) (18). After rating their distress, the patient is asked to identify one or many causes from a pre-established list of 39 potential items that commonly result in distress. These 39 items are categorized into 5 domains: practical problems; family problems; emotional problems; spiritual/religious concerns; physical problems. The tool has been translated into 21 languages, and 18 have validated the tool, including French (20). The biggest advantage of the Distress Thermometer compared to other tools is its ease of administration and interpretation, and brevity of use. Once screening with the Distress Thermometer is completed, the next decision is whether or not the user needs to be referred for psychosocial support. A barrier to using the Distress Thermometer is the ambiguity of the meaning of distress. In this study, a definition of distress will be provided to the user to minimize confusion. This definition is derived from the National Comprehensive Care Network Distress Management Panel, and will be modified to the following; “an unpleasant experience with a psychological, social, financial and/or physical nature that may interfere with your ability to cope effectively with the stressor” (19). Another barrier is the extensive list of potential sources that may not be relevant in non-oncologic users. We will reduce the number to 9 from the original 39 to those that are most relevant in this population. Another barrier is the variation associated between the distress rating and the need for referrals for help. In response, we will include an additional question after the user has completed the VAS and identified the potential cause(s) contributing to distress that asks, “Will your distress stop you or will your distress cause you to do something that would stop you from showing up for class or work today?”. This last question is intended to be used in conjunction with the distress rating to identify those users most at risk for absenteeism or drop-out.

## 11.12 Web-based, self-report tool for distress

The Distress Thermometer screening tool will be integrated into a web-based, self-reporting tool that apprentices will be asked to complete on a daily basis (Monday to Thursday) prior to attending their in-class or on-site session for the 12-week duration of the TDTP. Apprentices whose cohorts are randomized to either ICM^10^ or ICM^11^ will be provided with a tablet computer to access the Distress Thermometer screening tool. Apprentices will login using a unique username and password system. The Case Manager will have immediate access to each apprentice’s daily distress score, including all of their previous scores. The scores will be prioritized according to the following criteria:

1. High Priority Distress score ≥ 4 + Answer “Yes” to last question, or

Answer “Yes” to last question

A referral may also be made if the apprentice’s distress level is escalating defined as a change of ≥ 2 on the distress score from previous day.

2. Medium Priority Distress score≥4 + Answer “No” to last question

3. Low Priority Distress score ≤3 + Answer “No” to last question

The Case Manager will immediately contact (by phone) all apprentices in the high priority group to coordinate a care management plan personalized for their identified stressor(s). For those apprentices identified as medium priority, a one-on-one in-person meeting with the Case Manager will be scheduled to occur within 48 business hours in order to coordinate a personalized care management plan for their identified stressor(s). For those apprentices identified as low priority, a quick check-in with the Case Manager will take place within the work week to determine if any special care management issues need to be addressed. The care management plan is not dictated by the study but is left to the discretion of the Case Manager and apprentice. The care management plan will be documented within the web-based, self-report tool.

For those apprentices who do not compete a daily assessment by 8 am, the Case Manager will reach out by email/phone immediately to the apprentice to enquire about their status. For those apprentices experiencing severe distress, they have the option of directly contacting (email/phone) the Case Manager instead of completing the Distress Thermometer screening tool.

## 11.2 Mental health & addictions

When an apprentice has been identified as being in a high priority group due to a mental health or addictions stressor, the Case Manager will contact (by email or text message) the triage counsellor for the MH&A program intervention. After this initial contact, a standard referral form will be sent to the Case Manager at Community Builders to complete and return to the MH&A triage counsellor. The triage counsellor will review the referral form immediately and schedule an appointment with a MH&A healthcare provider. The timing of the appointment will be determined by the triage counsellor but will always occur within 24 to 48 hours of the referral. The triage counsellor will also determine the need for an in-person versus audio/video consultation.

For any apprentice identified as being at high risk for suicide, the triage counsellor will instruct the Case Manager to take the apprentice to the RVRHC Emergency Department where they will be assessed by the MH&A crisis team.

For any apprentice identified as being in either drug withdrawal or relapse, the triage counsellor will schedule an appointment with healthcare providers at the RVRHC Rapid Access Addiction Medical clinic. The appointment will always occur within 24 to 48 hours of the referral.

For any apprentice that requires treatment or admission to a MH&A program that limits their availability to complete the TDTP, they will be withdrawn from their cohort and enrolled in a subsequent TDTP cohort after resolution of their health-related issues. For all other apprentices who access MH&A services, they will continue to attend their TDTP and continue to be supported according to their allocated case management model.

## 11.3 Compliance

To promote study enrollment and adherence with study-related tasks, each enrolled apprentice will be provided with a tablet computer for the duration of the TDTP to support their participation and promote adherence to daily screening with the Distress Thermometer self-reporting tool. Apprentices who complete ≥ 90% of their daily Distress Thermometer screens will receive $50 for their time and effort and be enrolled in a draw for 5 tablet computers. The Apprentices enrolled in ICM^10^ and ICM^11^ do have the option of not completing the Distress Thermometer screening tool. These apprentices will be asked to relate the reason for each episode of non-compliance from a fixed list available in the self-report tool. Apprentices who choose not to complete the Distress Thermometer screening tool but document the reason will be considered to be compliant for that episode.

# 12 Outcomes

## 12.1 Primary outcome measures

### 12.11 Program attendance

Difference in proportion of absence-free program days (AFDs) at 12 weeks from the start date of the TDTP, where ***absence-free days are defined by the cumulative number of days of program attendance during the 12-week study period.*** The potential number of AFDs for each apprentice is the cumulative number of program days that the apprentice is alive during the 12-week TDTP, with a maximum of 48 days (=12 weeks x 4 days/week). A day is defined as an 8–10-hour work-day during the TDTP from Monday to Thursday. An apprentice is considered to have attended a work-day as long as the Case Manager or their construction supervisor documents their attendance.

### 12.12 Program completion

Difference in proportion of drop-outs at 12 weeks from start date of TDTP, where ***a drop-out is defined as an apprentice who fulfils any of the following criteria:***

- Has missed more than 50% of training days, or
- Who has elected to leave the program for reasons other than taking another job or returning to school

The proportion of drop-outs is defined as the ratio between the cumulative number of apprentices who meet the criteria for drop-out relative to the cumulative number of apprentices enrolled in the TDTP.

### 12.13 Post-program employment

Difference in proportion of full-time employment at 24-months post-TDTP completion, where ***full-time employment is defined as paid work ≥ 30 (median) hours per week at their main or only job.*** The reference period that will be used to determine full-time employment is the 4-week period preceding the 24-month post-TDTP completion date. The criteria for full-time employment will be considered to be met if the hours worked are reported as <30 hours per week for the following reasons: vacation; maternity; seasonal business; labour dispute; weather. Full-time employment will be self-reported by the TDTP graduate, and consent for corroboration with the employer will be requested by the study team. The proportion of full-time employment is defined as the ratio between the cumulative number of TDTP graduates who fulfill full-time employment criteria relative to the total number of TDTP graduates. A TDTP graduate is defined as any apprentice who successfully completed the TDTP (Section 12.12).

## 12.2 Secondary outcome measures

### 12.21 Mental Health & Addictions support

Difference in time to access mental health & addiction services, where ***time to access is defined as the difference (hours) between the date of referral from the Case Manager to the date of the MH&A appointment.***

### 12.22 Healthcare utilization

Difference in incidence rates of healthcare days at 12-weeks from the start date of the TDTP, where ***healthcare days represent the number of days alive and registered for an emergency room, mental health outpatient or addictions outpatient visit, or admitted to an acute care, mental health or detoxification facility.*** The incidence rate is defined by the ratio of the total number of healthcare days relative to the total person days exposure over the 12-week TDTP. The potential number of healthcare days for each apprentice is the number of days alive during the 12-week TDTP, with the maximum being 84 days (=12 weeks x 7 days/week).

### 12.23 Apprentice Satisfaction

Difference in apprenticeship TDTP satisfaction scores, where ***TDTP satisfaction scores will be measured upon TDTP completion using the validated National Centre for Vocational Education Research Student Outcomes Survey Satisfaction scores.*** Since 1995, this survey has been used to measure student satisfaction with vocational education and training (21) (Appendix). The survey consists of 19 individual questions divided into 3 major themes (Teaching, Assessment, and Generic skills and learned experiences) and 1 summary question. The response for each question is a Likert scale from *Strongly disagree (score=0)* to *Not Applicable (score=5).* The mean score for each theme and the score for the summary question will be used to estimate the differences in TDTP satisfaction. Only apprentices who successfully complete the TDTP will be asked to complete the survey.

### 12.24 Case Management Utilization

Difference in mean cumulative hours spent on case management during the TDTP by the Training Program Coordinator, where ***hours spent by the Training Program Coordinator on case management will be recorded prospectively by the Training Program Coordinator using the self-report tool.*** Case management-related activities represent all out-of-class activities that the Training Program Coordinator undertakes to individually support the apprentices during the TDTP. The Training Program Coordinator will record time commitments in 0.25 hours increments, rounded up to the nearest quarter hour. For example, if the Training Program Coordinator spends 20 minutes supporting an apprentice, they would record 0.5 hours case management-related activity.

### 12.25 Acceptability of self-report tool

To measure the acceptability of using the Distress Thermometer screening tool by apprentices and the Training Program Coordinator, where ***acceptability is measured using a 2-item questionnaire.*** The 2-item questionnaire was developed to measure acceptability of the Distress Thermometer tool among different user groups (22). The 2-items are:

1. Rate the degree of difficulty you encountered in using (monitoring) the Distress Thermometer screening tool.

The response options are: *Very easy (score=1); Somewhat easy; Somewhat hard; Very hard (score=4)*

2. To what extent did completing (monitoring) the Distress Thermometer screening tool bother you?

The response options are: *Didn’t bother me at all (score=1); Bothered me a little bit; Bothered me a lot (score=3)*

### 12.26 Feasibility of self-report tool

To measure the feasibility of using the Distress Thermometer screening tool by apprentices and the Training Program Coordinator, where ***feasibility is measured using a 1-item questionnaire.*** The 1-item questionnaire was developed to measure feasibility of the Distress Thermometer tool among different user groups (22). The 1-item questionnaire is:

1. How feasible was it to use (monitor) the Distress Thermometer on a daily basis?

The response options are: *Very feasible (score=1); Somewhat feasible; Somewhat infeasible; Very infeasible (score=4)*

### 12.27 Compliance of self-report tool

To measure apprentices’ compliance with the Distress Thermometer screening tool, where ***compliance is defined as the ratio of completed daily screens relative to the total number of TDTP days.*** The criteria for a completed daily screen include filling out the distress score, the source of distress, and the ‘yes/no’ question, or identifying the reason for not completing those items.

# 13 Participant timeline

| Activity | Study Period (Days) | | | | |
| --- | --- | --- | --- | --- | --- |
|  | T-1 | T0 | T1 | T2 | T3 |
|  | -7 | 0 | 1-60 (M-F) | 61-70 | 800 |
| **Enrollment** | | | | | |
| Cohort Randomization | x |  |  |  |  |
| Informed Consent |  | x |  |  |  |
| Demographics |  | x |  |  |  |
| Foundational skills survey |  | x |  |  |  |
| **Interventions** | | | | | |
| Distress Thermometer screening |  |  | x |  |  |
| MH&A program |  |  | x |  |  |
| **Assessments** | | | | | |
| TDTP Attendance |  |  | x |  |  |
| TTDP Completion |  |  |  | x |  |
| Employment |  |  |  |  | x |
| Time to Access MH&A |  |  |  | x |  |
| Healthcare utilization |  |  |  | x |  |
| TDTP Satisfaction survey |  |  |  | x |  |
| Case management utilization |  |  |  | x |  |
| Distress Thermometer tool |  |  |  |  |  |
| Acceptability |  |  |  | x |  |
| Feasibility |  |  |  | x |  |
| Compliance |  |  |  | x |  |

# 14 Sample size and power

All power estimations were conducted using nQuery version 8.7.2.0 (<https://www.statsols.com>; accessed June 10, 2022).

## 14.1 Difference in proportion of AFDs

The sample size is fixed at approximately 448 individuals. We anticipate that the first few cohorts will be asked to pilot the screening tool, potentially removing up to 50 apprentices from the final sample size. We will use a fixed sample size of 400 apprentices, divided equally between Sudbury and Barrie sites. The expected cohort sizes will range from 6 to 10 apprentices, resulting in 20 to 34 cohorts per site. We will use a 3-level hierarchical design to model differences in proportion of AFDs where the daily attendance (Level 1) will be coded as binary; 0 for absence and 1 for attendance. Level 1 observations are clustered within individual apprentices (Level 2), which are nested within individual cohorts (Level 3). The sites (Sudbury and Barrie) and start date of cohort enrollment (q1 (January-March); q2 (April-June); q3 (July-Sept); q4 (Oct-Dec) and year) will be included as confounding variables in Level 1 and will not enter into the power calculations. Using this design, we performed a sensitivity analysis to determine the power to observe different primary outcome effect sizes with an $\alpha$-level 0.05**.**  As can be seen in the following table, even small differences in attendance (5% difference in proportions of AFDs) would still be detected with power ≥ 80% as long as the Level 2 inter-cluster correlation ≤ 0.125, a very reasonable assumption as there is no reason to believe that these apprentices’ attendance behaviours will be highly correlated with each other (23).

|  | **Scenarios**^1^ | | | | | | | |
| --- | --- | --- | --- | --- | --- | --- | --- | --- |
| **Variables** | 1 | 2 | 3 | 4 | 5 | 6 | 7 | 8 |
| Test Significance Level, α​ | 0.05 | 0.05 | 0.05 | 0.05 | 0.05 | 0.05 | 0.05 | 0.05 |
| Mean Difference, δ = µ₁ - µ₂​ | 0.1 | 0.15 | 0.05 | 0.05 | 0.05 | 0.05 | 0.05 | 0.1 |
| Standard Deviation, σ​ | 0.1 | 0.1 | 0.1 | 0.1 | 0.1 | 0.1 | 0.15 | 0.15 |
| Level 1 Unit Correlation, ρ₁​ | 0.75 | 0.75 | 0.75 | 0.75 | 0.75 | 0.75 | 0.75 | 0.75 |
| Level 2 Unit Correlation, ρ₂​ | 0.5 | 0.5 | 0.5 | 0.25 | 0.125 | 0.05 | 0.05 | 0.05 |
| Number of Level 3 Units in Group 1, C₁​ | 15 | 15 | 15 | 15 | 15 | 15 | 15 | 15 |
| Number of Level 3 Units in Group 2, C₂​ | 15 | 15 | 15 | 15 | 15 | 15 | 15 | 15 |
| Level 3 Units per Group Ratio, C₂/C₁​ | 1 | 1 | 1 | 1 | 1 | 1 | 1 | 1 |
| Number of Level 2 Units per Level 3 Unit, K​ | 6 | 6 | 6 | 6 | 6 | 6 | 6 | 6 |
| Number of Level 1 Units per Level 2 Unit, M​ | 10 | 10 | 10 | 10 | 10 | 10 | 10 | 10 |
| Power (%)​ | 95.9 | 99 | 45.7 | 65.4 | 80.9 | 91.1 | 59.8 | 99 |

^1^ Highlighted cells (Yellow) represent changes from previous scenario

## 14.2 Difference in proportions of TDTP completion

Historical cohorts have demonstrated variation in program completion proportions but have been generally observed at a 75-80% graduation / completion rate. In this cluster randomized trial comparing the difference in proportions of TDTP completion, we define an *a priori* difference of ≥ 0.1 in completion proportions as the lower limit of a minimally significant difference (24). This is opinion-based on an informal survey of TDTP stakeholders. Given the same fixed sample and cohort size estimates discussed in Section 14.1, we performed a sensitivity analysis to determine the power to observe a difference in program completion proportions ≥ 0.1 using different control group completion rates with an $\alpha$-level 0.05**.**  As can be seen in the following table, a difference in completion proportions of 0.1 would be detected with power ≥ 80% as long as the control group completion proportion was ≥ 0.8, a reasonable assumption given the historically observed program completion proportions.

|  | **Scenarios**^1^ | | | | | | |
| --- | --- | --- | --- | --- | --- | --- | --- |
| **Variables** | 1 | 2 | 3 | 4 | 5 | 6 | 7 |
| Test Significance Level, α​ | 0.05 | 0.05 | 0.05 | 0.05 | 0.05 | 0.05 | 0.05 |
| 1 or 2-Sided Test?​ | Two | Two | Two | Two | Two | Two | Two |
| Control Group Proportion (p2)​ | 0.75 | 0.75 | 0.75 | 0.75 | 0.8 | 0.8 | 0.8 |
| Test Statistic under H0​ | 0 | 0 | 0 | 0 | 0 | 0 | 0 |
| Test Statistic under H1​ | 0.1 | 0.1 | 0.1 | 0.1 | 0.1 | 0.1 | 0.1 |
| Intracluster Correlation (ICC)​ | 0 | 0.05 | 0 | 0 | 0 | 0 | 0 |
| Clusters in Treatment Group (K1)​ | 20 | 20 | 33 | 25 | 20 | 33 | 25 |
| Clusters in Control Group (K2)​ | 20 | 20 | 33 | 25 | 20 | 33 | 25 |
| Cluster Ratio (K2/K1)​ | 1 | 1 | 1 | 1 | 1 | 1 | 1 |
| Cluster Sample Size in Treatment Group (M1)​ | 10 | 10 | 6 | 8 | 10 | 6 | 8 |
| Cluster Sample Size in Control Group (M2)​ | 10 | 10 | 6 | 8 | 10 | 6 | 8 |
| Cluster Sample Size Ratio (M2/M1)​ | 1 | 1 | 1 | 1 | 1 | 1 | 1 |
| Power (%)​ | 70.6 | 54.6 | 70.2 | 70.6 | 80.2 | 79.8 | 80.2 |

^1^ Highlighted cells (Yellow) represent changes from previous scenario

## 14.3 Difference in proportions of full-time employment

Differences in full-time employment proportions between the groups is expected to be similar to differences in proportions of TDTP program completion.

# 15 Recruitment

To be eligible for TDTP enrollment, individuals must be Canadian, female, black, from a racialized community, an immigrant, and/or Indigenous. Eligible individuals must submit to an interview with Community Builders®. Successful candidates must demonstrate that they are a good fit for the TDTP by showing, at a minimum, an interest in the construction trades and possessing some basic English or French-speaking and writing skills. Enrollment in the TDTP is not contingent on eligible individuals agreeing to participate in the TeachMeToBuild study. Individuals enrolled in the TDTP whose cohort is randomized to the Distress Thermometer screening tool intervention will be introduced to the TeachMeToBuild study during their initial TDTP orientation. The case managers will use the expressed consent powerpoint presentation to introduce the study to the students. During this introduction, all TDTP students will be informed that their participation in the study is voluntary and refusal to enrol or withdraw after study enrollment will not affect their subsequent TDTP participation or outcomes. After this introduction, each individual will be provided with the contact information (email and telephone number) for a study team member that can be reached to further discuss the study and possible enrollment. For those individuals who contact the study team member, an in-person meeting or audio/video teleconference will be scheduled. During this meeting, the study team member will discuss the study rationale, objectives, participant responsibilities, data collection, risks and benefits. During this meeting, the study team member will encourage and answer study-related questions. For interested individuals, the study team member will review the informed consent form (ICF) and answer all questions. Interested individuals will be provided with a paper/electronic copy of the ICF to review, and a subsequent follow-up in-person or audio/video teleconference meeting in 72 hours (business days) will be scheduled between the study team member and the interested individual to determine their interest to enroll in the TeachMeToBuild study. All TDTP students will be reminded by the study team member during the meetings that study participation is voluntary and refusal to enrol or withdraw after study enrollment will not adversely affect their subsequent TDTP participation or outcomes. To withdraw from the study, the participant will inform the case manager who will then inform the study team members.

Potential study participants will be offered several incentives to enroll in the study. Each study participant will be provided with a tablet computer for the duration of the 12-week TDTP to facilitate their daily entries in the Distress Thermometer screening tool. These tablet computers will be provided free-of-charge. Study participants will not be responsible for the costs associated with any lost, stolen or damaged units. These tablet computers must be returned to Community Builders® at the end of the 12-week TDTP. Study participants who complete ≥ 90% of their daily Distress Thermometer screens will receive $50 for their time and effort and be enrolled in a draw for 5 tablet computers to be awarded at the end of the TeachMeToBuild study.

# 16 Allocation

## 16.1 Sequence generation

Cohorts will be randomly assigned to either BCM or ICM^1x^ (Figure 1) with a 1:1:1:1 allocation schema as per a computer-generated randomisation schedule stratified by site using permuted blocks of size 4 in each strata. A single block size was chosen to accommodate potentially different cohort numbers resulting from variation in the cohort sample sizes. The block size will not be disclosed to ensure concealment.

## 16.2 Concealment mechanism

The random allocation sequence will be generated using the *ralloc* function in STATA 17/MP for Mac. This allocation sequence will be uploaded into the Royal Victoria Regional Health Centre Research Electronic Database Capture (REDCap®) system (25,26), which is an online, password-protected, web-based research electronic database system stored on the Personal Health Information Protection Act (PHIPA) (27)-protected servers at the Royal Victoria Regional Health Centre. An independent study coordinator not associated with the TDTP will be responsible for accessing the password-protected allocation sequence from REDCap®. Only the study coordinator will have access to the allocation sequence to avoid any influence from the study investigators, TDTP Training Program Coordinator, or other personnel. Allocation concealment will be ensured as the study coordinator will not release the randomisation code until each cohort has completed registration of all of its TDTP students.

## 16.3 Implementation

Cohort randomization will be requested by the TDTP Training Program Coordinator from the independent study coordinator (Section 16.2) 1 week prior to that cohort’s TDTP start date by calling a centralized study telephone number. The study coordinator will access the random allocation sequence through REDCap®, and then inform the Training Program Coordinator of the allocation for the cohort. The Training Program Coordinator will provide this allocation information to the TDTP students. Informed consent from each interested TDTP student in the randomized cohorts ICM^1x^ will then be obtained by *TeachMeToBuild* study personnel (Section 15).

# 17 Masking

This is an open-label, pragmatic study so neither study participants nor Community Builders® nor study personnel, including data analysts, will be masked to the allocation.

# 18 Data Collection

## 18.1 Methods

All demographic and study-related data inputted into the web-based Distress Thermometer screening tool by TDTP students and personnel will be encrypted for transfer and storage in a Microsoft Azure SQL Database. None of the data related to the Distress Thermometer screening tool will be stored locally on the tablet computer. All routinely collected data during clinical encounters will be collected by the MH&A healthcare providers and stored in MEDITECH Expanse, the electronic health record system used at the Royal Victoria Regional Health Centre. Any other study-specific data will be collected by study personnel and stored in electronic case report forms created in REDCap®.

### 18.11 Distress Thermometer data

**All** data variables will contain an option in the drop-down list that states “Rather not say”

| Data | Description | Data type |
| --- | --- | --- |
| **Demographics** | | |
| Age | Years | Integer |
| Date of birth | mmddyy | Continuous |
| Gender identification | Man (cis & transgender) | Categorical |
|  | Woman (cis and transgender) |  |
|  | Non-binary (neither solely man nor woman) |  |
| Country of birth | Canadian-born | Categorical |
|  | Foreign-born |  |
| Education | Below high school graduation | Categorical |
|  | High school diploma |  |
|  | Trades (includes certification or diploma from vocational school or apprenticeship training) |  |
|  | College (includes non-university certificate or diploma from community college, CEGEP, school of nursing and similar programs, university certificate below bachelor’s level) |  |
|  | University (includes bachelor’s degree and university degree or certificate above bachelor’s degree) |  |
| Ethnic/Cultural origin | North American Indigenous | Categorical |
|  | Other North American |  |
|  | European |  |
|  | Caribbean |  |
|  | Latin, Central, South America |  |
|  | African |  |
|  | Asian |  |
|  | Oceania |  |
| Visible minority | Visible minority (persons, other than Indigenous peoples, who are non-Caucasian in race or non-white in colour) | Categorical |
|  | Not a visible minority |  |
| Indigenous group | First Nations | Categorical |
|  | Status |  |
|  | Non-status |  |
|  | Métis |  |
|  | Inuk/Inuit |  |
| Children | None | Categorical |
|  | 1 |  |
|  | 2 |  |
|  | >2 |  |
| In the last **10 years**, have you had: | | |
| Criminal offence | Yes | Categorical |
|  | No |  |
| Mental health disorder | Yes | Categorical |
|  | No |  |
| Substance use disorder | Yes | Categorical |
|  | No |  |
| **Distress Thermometer** | | |
| Visual analog scale | 0-10 | Integer |
| Distress sources | Mental health | Categorical |
|  | Substance use/cravings |  |
|  | Family/Relationships |  |
|  | Financial |  |
|  | Physical health |  |
|  | Work program |  |
|  | Housing |  |
|  | Change in medication |  |
|  | Legal Issues |  |
| Distress question | Yes | Categorical |
|  | No |  |
| **Case Manager** | | |
| Action | Case Manager counselling/support | Categorical |
|  | External service consult (not related to MH&A) |  |
|  | External service consult (Mental health) |  |
|  | External service consult (Addictions) |  |
| Date/Time (Action) | ddmmyy:hh | Continuous |
| Case management time | 0.15 hr units | Continuous |
| Apprentice attendance (Daily (M-F) for 12-week TDTP) | Yes | Categorical |
|  | No |  |
|  |  |  |

### 18.12 Routinely collected data

Any clinical encounters with a healthcare provider for any reason may require the collection of personal health information, such as Ontario Health Insurance Plan number, along with other information as dictated by the healthcare provider, healthcare facility, and diagnostic and therapeutic care plan. The PHIPA-compliant storage and transmission of this routinely collected clinical data will be the responsibility of the health information custodian. This study has no requirements for the *a priori* collection of any routinely collected clinical data.

Other routinely collected data specific to the TDTP includes daily attendance and program completion. The Training Program Coordinator is responsible for this data collection. The daily attendance data will be collected using the Distress Thermometer screening tool. Program completion data will be collected at the end of the 12-week TDTP and entered into the electronic case report form in REDCap by study personnel.

### 18.13 Study-specific data

| Data | Description | Data type |
| --- | --- | --- |
| **Cluster criteria** |  |  |
| City | Barrie vs Sudbury | Categorical |
| Enrollment | Quarter-year  Quarter (Q):  Q1=January 1 – March 31  Q2=April 1 – June 30  Q3=July 1 – September 30  Q4=October 1 – December 31 | Categorical |
| **Primary Outcomes** |  |  |
| Program completion | Yes | Categorical |
|  | No |  |
| Employment (full-time at 24 months) |  | Categorical |
| Self-report | Yes | Categorical |
|  | Employer verified |  |
|  | Employer not verified |  |
|  | No |  |
| **Secondary Outcomes** |  |  |
| Date/Time |  | Continuous |
| Mental health consult | ddmmyy:hh |  |
| Addictions consult |  |  |
| TDTP Satisfaction Survey | National Centre for Vocational Education Research Student Outcomes Survey (21) | Categorical |
| Distress Thermometer |  |  |
| Acceptability survey | Section 12.25 | Categorical |
| Feasibility survey | Section 12.26 | Categorical |

#### 18.13a Foundational skills

Complete essential skills self-assessment for the trades questionnaire (28). There are 9 foundational skills identified in the questionnaire, each containing statements that require the apprentice to answer *yes, somewhat or no.*  For any skills sub-domain in which the apprentice answers *somewhat* and/or *no* in less than 5 statements, the apprentice is considered to possess a *strength* in that foundational skill. A cumulative score of the number of foundational skills that each apprentice possesses (out of 9) will be calculated and used as a baseline variable. Each apprentice will be asked to complete this survey as part of the routine orientation process. The survey link will be provided to each apprentice and all results will be stored in REDCap using a unique identifier number.

### 18.14 Healthcare utilization

This data (see Section 12.22) is available from the Registered Persons Database, Ontario Mental Health Reporting System, Discharge Abstract Database, and National Ambulatory Care Reporting System through the Institute for Clinical Evaluative Sciences using a data linkage with each patient’s unique Ontario Health Insurance Plan number.

As a prescribed entity under the Personal Health Information Protection Act (27), the Institute for Clinical Evaluative Sciences is authorized to collect personal health information from health organizations without consent for the purposes of evaluation and monitoring of Ontario’s health system. The Institute for Clinical Evaluative Sciences is prohibited, under its agreements

with data providers, from contacting individuals whose information has been

entrusted to the Institute for Clinical Evaluative Sciences. This contractual obligation restricts any opportunity to seek individuals’ consent for use of their information for research.

The Institute for Clinical Evaluative Sciences will make available a research-ready, linked and risk-reduced coded dataset to the study investigators. Although highly de-sensitized, the research data is presented at an individual level. Study investigators will access the Research Data remotely on a secure, encrypted VMware virtual desktop called the Institute for Clinical Evaluative Sciences Data & Analytic Virtual Environment (IDAVE). Study investigators will perform analyses on IDAVE using statistical software. Research Data may not be copied or transferred from IDAVE. Only results derived from the Research Data that have been vetted for

re-identification risk and approved by Institute for Clinical Evaluative Sciences may be released from IDAVE.

## 18.2 Retention

### 18.21 Strategies

Once an apprentice is enrolled in the TDTP, the Training Program Coordinator will make every reasonable effort to develop a BCM plan for each apprentice that will be directed at all the existing or potential barriers that may threaten their program attendance or completion.

### 18.22 Withdrawal

Apprentices may choose to withdraw from the study for any reason at any time without any adverse consequences on their TDTP enrollment. For those apprentices who withdraw for any reason, all data collected up to the time of their withdrawal will be included in the final analysis. Deviations from the study protocol (which do not result in withdrawal) or loss-to-follow-up for any reason will not be considered reasons for withdrawal from the study.

# 19 Data management

All data collected using the Distress Thermometer screening tool (Section 18.11) will be encrypted and stored in a Microsoft Azure SQL Database. Differential backups of the database will be performed automatically once every 24 hours, with full backups being performed automatically once every week. Backups are replicated to three separate Azure data centers within the same region, ensuring that data will not be lost if one data center becomes unavailable. All instances where personal health information is created, viewed, or updated will be recorded in an electronic audit trail that identifies the person to whom the information relates, the user accessing the information, the type of information, and the date and time that the information was accessed. Data access by users of the tool is regulated by privileges associated with their user identification and password. The data is password-protected and will only be accessible by apprentices during their 12-week TDTP after which time they will no longer be able to access the screening tool. In addition, the TDTP Training Coordinator will have password-protected access to all apprentices’ data for their 12-week TDTP after which time they will no longer be able to access those apprentices’ data. The MH&A counsellors will have password-protected access to a limited data set that includes only those apprentices who have had a clinical referral. Again, access will be limited to the 12-week TDTP.

All demographic data (Section 18.11) will be entered electronically by apprentices into REDCap through a secure survey link that will be sent by email upon registration in the Distress Thermometer screening tool. A unique study number will be assigned to each apprentice in REDCap. This unique study number, along with the patient’s name (first, middle, last), Ontario Health Insurance Plan number, date of birth, and unique electronic health record system number (if applicable) will be stored in a password-protected EXCEL computer file. This study EXCEL file will be stored in a dedicated, password-protected electronic shared drive located on the Royal Victoria Regional Health Centre Personal Health Information Protection Act (PHIPA)-compliant servers. This EXCEL file will permit linkage to the electronic case report form to enable study personnel to record outcome data over the study period for a participant. Real-time data quality rules will be implemented in REDCap that will display warning pop-up messages whenever the rules are violated during data entry. These quality rules will minimize missing values in required fields; prevent incorrect data type entry and out of range data entry; identify outliers for numerical fields; and prevent invalid data entry into multiple choice fields. The data quality rules will also be available to be executed at any time by a study monitor or study personnel. All electronic case report form entries and edits are associated with an electronic audit trail that identifies the user, date and time of entry, and entry type. The type of activity that study personnel may undertake in REDCap is regulated by privileges associated with their user identification and password. Incremental data back-ups of REDCap are routinely performed twice a day, with off-site storage of the backed-up files done on a monthly basis.

All study-specific data that is survey-based (Section 18.13 and 18.13a) will be completed by apprentices during orientation or after the completion of the TDTP. Secure links for each of the surveys will be sent by email to each apprentice. All survey responses will be stored in REDCap using the previously assigned unique identifier. Study-specific data related to clinical outcomes will be extracted by study personnel from the electronic medical record system (dates and times of Mental Health and/or Addictions healthcare visits) and stored in REDCap. All other study-specific outcome data related to program completion and employment (Section 18.13) will be collected by study personnel who will enter the data in REDCap.

All study data will be archived in REDCap for 10 years and subsequently permanently destroyed.

# 20 Statistical methods

## 20.1 Outcomes

The intervention arms (ICM^10^ (Distress Thermometer screening tool), ICM^01^ (MH&A fit-for-purpose program), ICM^11^ (Both Distress Thermometer and MH&A) will be compared against the active comparator (BCM) for all primary and secondary analyses where applicable. All inferential and descriptive analyses will be at the individual level after accounting for the effects of clustering (id and cohort).

### 20.11 Primary

#### 20.11a Program attendance

We will use a 3-level hierarchical design to model the program attendance data where the observation for each day (Level 1) will be coded as binary; attendance (=1) versus absence (=0). Level 1 observations are clustered within individual apprentices (Level 2) , which are nested within cohorts (Level 3). We will analyse the outcome data using multi-level, mixed-effects logistic regression analysis with fixed effects estimated for the variance in the intercepts of both apprentice and cohort levels. The interventions (Distress thermometer and MH&A) will be included as well as a variable for their interaction effect. We will also include time period (year-quarter) and an interaction term between the time period and each intervention (Distress thermometer and MH&A). A sensitivity analysis will be conducted to compare this baseline model with an extended model that includes any baseline demographic or clinical variables that appear to be unbalanced at Level 1. The models will be compared using the likelihood-ratio comparison test for superiority. Post-regression analyses using *margins* command will be used to estimate difference in program attendance between treatment groups.

#### 20.11b Program completion

We will use a logistic multivariate regression analysis to model program completion where the observation for program completion will be coded as binary: completion (=1) and drop-out (=0). The interventions (Distress thermometer and MH&A) will be included as well as a variable for their interaction effect. We will also include time period (year-quarter) and an interaction term between the time period and each intervention (Distress thermometer and MH&A). Clustered robust standard errors will be used to account for cohort intra-cluster correlation. Post-regression analyses using *margins* command will be used to estimate difference in program completion between treatment groups.

#### 20.11c Post-program employment

We will use a logistic multivariate regression analysis to model post-program employment where the observation for post-program employment will be coded as binary: full-time (=1) and not full-time (=0). The interventions (Distress thermometer and MH&A) will be included as well as a variable for their interaction effect. We will also include time period (year-quarter) and an interaction term between the time period and each intervention (Distress thermometer and MH&A). Clustered robust standard errors will be used to account for cohort intra-cluster correlation. Post-regression analyses using *margins* command will be used to estimate difference in post-program employment between treatment groups.

### 20.12 Secondary

#### 20.12a Mental Health & Addictions support

We will use a 3-level hierarchical design to model the time difference to access MH&A support data. Level 1 observations are clustered within individual apprentices (Level 2) , which are nested within cohorts (Level 3). We will analyse the outcome data using multi-level, mixed-effects linear regression analysis with fixed effects estimated for the variance in the intercepts of both apprentice and cohort levels. The interventions (Distress thermometer and MH&A) will be included as well as a variable for their interaction effect. We will also include time period (year-quarter) and an interaction term between the time period and each intervention (Distress thermometer and MH&A). A sensitivity analysis will be conducted to compare this baseline model with an extended model that includes any baseline demographic or clinical variables that appear to be unbalanced at Level 1. The models will be compared using the likelihood-ratio comparison test for superiority. Post-regression analyses using *margins* command will be used to estimate the time difference to access MH&A support between treatment groups.

#### 20.12b Healthcare utilization

We will model the days of healthcare utilization as count data, with the period of follow-up while alive during the study period as the exposure period. We will analyse the data using a Poisson multivariate regression model. The interventions (Distress thermometer and MH&A) will be included as well as a variable for their interaction effect. We will also include time period (year-quarter) and an interaction term between the time period and each intervention (Distress thermometer and MH&A). Clustered robust standard errors will be used to account for clustering of data within apprentices.

#### 20.12c Apprentice satisfaction

We will use linear multivariate regression analysis to model the differences in mean scores for each domain (3 major themes and summary question) of the National Centre for Vocational Education Research Student Outcomes Satisfaction Survey. The interventions (Distress thermometer and MH&A) will be included as well as a variable for their interaction effect. We will also include time period (year-quarter) and an interaction term between the time period and each intervention (Distress thermometer and MH&A).

#### 20.12d Case management utilization

We will use linear multivariate regression analysis to model the mean difference in cumulative hours spent on case management by the Training Program Coordinator. The interventions (Distress thermometer and MH&A) will be included as well as a variable for their interaction effect. We will also include time period (year-quarter) and an interaction term between the time period and each intervention (Distress thermometer and MH&A).

#### 20.12e Acceptability and feasibility of self-report tool

We will use linear multivariate regression analysis to model the difference in mean scores for each question in both the acceptability and feasibility scales. The interventions (Distress thermometer and MH&A) will be included as well as a variable for their interaction effect. We will also include time period (year-quarter) and an interaction term between the time period and each intervention (Distress thermometer and MH&A).

#### 20.12f Compliance of self-report tool

We will use linear multivariate regression analysis to model the difference in mean compliance rates for the Distress Thermometer self-report tool. The interventions (Distress thermometer and MH&A) will be included as well as a variable for their interaction effect. We will also include time period (year-quarter) and an interaction term between the time period and each intervention (Distress thermometer and MH&A).

# 21 Monitoring

There will be no data management or trial management committee, interim analysis or premature termination of the study.

# 22 Harms

Adverse events that are anticipated among this study population include, but are not limited to, program drop-out, failure to satisfy the criteria for program completion, ill health or hospitalizations due to substance abuse and mental health crises. These events will not be reported to the Research Ethics Board unless the investigators feel that the event may have been caused by the protocol procedure.

# 23 Auditing

## 23.1 Investigator responsibilities

The investigators agree to perform the clinical trial in accordance with this clinical trial protocol, International Council for Harmonisation guideline for Good Clinical Practice (<https://ichgcp.net/>) and all applicable regulatory requirements.

## 23.2 Coordinating centre responsibilities

The RVH Research Institute will be responsible for taking all reasonable steps to ensure proper conduct of the clinical trial protocol.

## 23.3 Site initiation

Prior to the initiation of the study at each study site, the RVH Research Institute will be responsible for providing adequate training to the case managers, healthcare providers and study personnel. The training will cover all aspects of the study protocol and procedures and will include practical training on the use of the randomisation system, electronic case report forms and study materials such as the Distress Thermometer. The site initiation visit will be conducted by either teleconference, video conference or face-to-face meetings at the participating study site. Written and electronic materials will be supplied for study personnel and for the education of the case managers and healthcare providers at each site.

## 23.4 Study monitoring

An independent study monitor from the RVH Research Institute will visit each participating site biannually during the study period. This will ensure that the study is conducted according to the protocol, good clinic practice guidelines and relevant regulatory requirements. The main duty of the study monitor is to help the principal investigators and the RVH Research Institute maintain a high level of ethical, scientific, technical and regulatory quality throughout all aspects of the trial. The principal investigators, healthcare providers, case managers and study personnel will assist the study monitor by providing all appropriate documentation and being available to discuss the study.

## 23.5 Site close-out

At the completion of the trial, a final monitoring and close out visit will be conducted by the study monitor.

## 23.6 Source documents

The study monitor will check the source documents to confirm the existence of the participant and the integrity of the study data. Source documents refer to the Distress Thermometer self-report tool used by the case managers, and any electronic medical records of the participants used by the healthcare providers during episodes of care related to the study. Adequate and accurate source documents allow the principal investigators and the study monitor to verify the reliability and authenticity of data recorded on the electronic case report forms and ultimately to validate that the study was carried out in accordance with the protocol.

## 23.7 Direct access to data and documents

The study may be audited by the RVH Research Ethics Board, or qualified representatives of the RVH Research Institute as permitted by regulations. Therefore, access to medical records, other source documents and other study related files will be made available at all study sites for monitoring and audit purposes during the study and after its completion.

Participants will not be identified by name, and confidentiality of information in medical records will be preserved. The confidentiality of the participant will be maintained unless disclosure is required by regulations.

# 24 Ethics approval

This protocol and appendices will be reviewed and approved by the RVH Research Institute and the Clinical Trials Ontario-accredited RVH Research Ethics Board with respect to scientific content and compliance with applicable research and human subjects’ regulations.

The protocol, informed consent form, participant education and recruitment materials, and other requested documents, and any subsequent modifications, also will be reviewed and approved by the RVH Research Ethics Board.

Subsequent to initial review and approval, the RVH Research Ethics Board will review the protocol at least annually. The Principal investigators will make progress reports to the RVH Research Ethics Board at least annually and within three months of study termination. These reports will include the total number of participants enrolled, completed the study, in follow-up, lost-to-follow-up, and withdrawn; protocol and informed consent deviations and modifications; and if applicable, total number of adverse events.

# 25 Protocol amendments

Any modifications to the protocol which may impact on the conduct of the study, potential benefit of the participant or may affect participant safety, including changes of study objectives, study design, participant population, sample sizes, study procedures, or significant administrative aspects will require a formal amendment to the protocol. Such amendments will be agreed upon by the Principal investigators, the RVH Research Institute, and approved by the RVH Research Ethics Board prior to implementation.

Administrative changes of the protocol are minor corrections and/or clarifications that have no effect on the way the study is to be conducted. These administrative changes will be agreed upon by the Principal investigators, the RVH Research Institute, and will be documented in a Note to File to the RVH Research Ethics Board.

# 26 Consent

This study fulfils the criteria for a low-risk intervention trial in that the study interventions and study-related assessments and follow-up pose no more than minimal additional risk or burden to the safety of the participants. As such, all eligible participants will be briefly informed by their case managers about the main features of the trial during their TDTP orientation session. This introduction will include information about randomisation, the Distress Thermometer self-report tool, support pathways for MH&A, study-related questionnaires and follow-up. For those participants who are interested in learning more about the study, they will be provided with the contact information for study personnel (Section 15). After contacting the study personnel, the informed consent process will be administered as described previously in Section 15.

# 27 Confidentiality

All participant data pertaining to the study will be stored in a computer database maintaining confidentiality in accordance with PHIPA regarding privacy and use of health data. When archiving or processing data pertaining to the investigators and/or to the participants, the RVH Research Institute will take all appropriate measures to safeguard and prevent access to this data by any unauthorized third party.

The investigators will maintain the confidentiality of all study documentation and take measures to prevent accidental or premature destruction of these documents. After the completion or discontinuation of the study the investigators will retain the study documents

ten (10) years in Canada as required by regulation. The investigators must notify the RVH Research Institute prior to destroying any study documents following study completion or discontinuation. If the investigators’ situation is such that archiving can no longer be ensured, the investigators will inform the RVH Research Institute, and the relevant records will be transferred to a mutually agreed designee.

If any of the investigators retire, relocate, or otherwise withdraw from conducting the study, the responsibility for maintaining records may be transferred to the RVH Research Institute or another investigator. The RVH Research Institute must be notified of and agree to the change. All associated documentation must also be updated.

# 28 Declaration of interests

GD and CS have never received any salary support or grants, honoraria, paid consultancies or service on advisory boards, receipt of patents or patents pending, ownership of stocks or options from Community Builders Construction or Alectra. SB is a paid employee of Community Builders Construction. Community Builders Construction has received a Federal grant from the Youth Employment and Skills Strategy program.

# 29 Access to data

The RVH Research Institute will oversee the intra-study data sharing process. The Principal Investigators will be given access to the cleaned data sets. Study data sets will be housed on the RVH REDCap web-site created for the study, and all data sets will be password protected. The Principal Investigators will have direct access to the data sets. To ensure confidentiality, any data dispersed to the study healthcare providers, case managers or study personnel will be de-identified participant information and aggregated whenever feasible.

# 30 Post-trial care

Should this study provide evidence of the superior effectiveness of using the Distress Thermometer and/or MH&A care pathways compared to usual case management, subsequent TDTP or similar construction vocational programs administered by Community Builders Construction will incorporate similar case management strategies.

# 31 Trial results and authorship

The study will be conducted in the name of the ‘TeachMeToBuild Trial Investigators’. Overall project coordination and data management will be provided by the RVH Research Institute. Study results will be disseminated via abstracts, trial registry, journal publication and RVH Research Institute and Community Builders Construction and Alectra websites regardless of the magnitude or direction of effect.

Authorship of publications arising from the study will be consistent with current International Committee of Medical Journal Editors’ recommendations (<http://www.icmje.org/recommendations/>) with full credit assigned to all collaborating investigators, healthcare providers, study personnel and corporations. Responsibility for the content of manuscripts will rest with the Principal Investigators, and where listed, Shawna Bailey will be listed as first author, Carrie Stoner will be listed as second author, and Giulio DiDiodato will be listed as corresponding author, with all other subsequent members listed alphabetically following Carrie Stoner and preceding Giulio DiDiodato.

Funding bodies will be acknowledged in all publications.

# 32 Reproducible research

The trial protocol, full study report, de-identified participant-level dataset, and statistical code for generating the results will be made publicly available no later than 3 years after study closure to an appropriate data archive for sharing purposes.

# 33 References

1. Morissette R. Chapter 2: Youth employment in Canada [Internet]. 2021. Available from: https://www150.statcan.gc.ca/n1/en/catalogue/42280001202100100002

2. Canadian Council on Social Development. Unequal access: A Canadian profile of racial differences in education, employment and income [Internet]. 2000. Available from: https://www.crrf-fcrr.ca/images/stories/pdf/unequal/Engfin.pdf

3. Expert Panel on Youth Employment. 13 Ways to Modernize Youth Employment in Canada: Strategies for a New World of Work [Internet]. 2017. Available from: https://www.canada.ca/content/dam/canada/employment-social-development/corporate/youth-expert-panel/report-modern-strategies-youth-employment/modern-strategies-youth-employment-en.pdf

4. Canadian Council of Directors of Apprenticeship. Red Seal Trades. 2022.

5. Standing Committee on Finance. Youth employment in Canada: Challenges and Potential Solutions [Internet]. Ottawa; 2014. Available from: https://publications.gc.ca/collections/collection_2014/parl/xc26-1/XC26-1-1-412-6-eng.pdf

6. Frank K, Frenette M. How Do Women in Male-dominated Apprenticeships Fare in the Labour Market? Anal Stud Branch Res Pap Ser. 2019;(Catalogue no. 11F0019M — No. 420):1–25.

7. Card D, Kluve J, Weber A. What Works? A Meta Analysis of Recent Active Labor Market Program Evaluations. J Eur Econ Assoc [Internet]. 2018 Jun 1;16(3):894–931. Available from: https://doi.org/10.1093/jeea/jvx028

8. Registered Apprenticeship Information System (RAIS). Canadian Apprenticeship Registrations and Certifications [Internet]. Statistics Canada. 2020 [cited 2022 May 31]. Available from: https://www150.statcan.gc.ca/n1/pub/71-607-x/71-607-x2020016-eng.htm

9. Statistics Canada. Median employment income of apprentices who certify in select trades two and five years after certification [Internet]. 2015 [cited 2022 May 31]. Available from: https://www150.statcan.gc.ca/t1/tbl1/en/tv.action?pid=3710001601&pickMembers%5B0%5D=1.7&pickMembers%5B1%5D=2.3&cubeTimeFrame.startYear=2013&cubeTimeFrame.endYear=2015&referencePeriods=20130101%2C20150101

10. Statistics Canada. The impact of COVID-19 on apprencticeship programs in Canada [Internet]. Apprentice Demand Across Canada. 2021 [cited 2022 May 31]. Available from: https://www150.statcan.gc.ca/n1/pub/11-627-m/11-627-m2021072-eng.htm

11. Palameta B, Myers K, Gyarmati D, Voyer J-P. Understanding training program effectiveness : A comprehensive framework Learning and Active Employment Programs Project [Internet]. Ottawa; 2011. Available from: https://www.srdc.org/media/10083/laep1_en.pdf

12. Bruns EJ, Pullmann MD, Sather A, Brinson RD, Ramey M. Effectiveness of Wraparound Versus Case Management for Children and Adolescents: Results of a Randomized Study. Adm Policy Ment Heal Ment Heal Serv Res. 2015;42(3):309–22.

13. Guarino K. Step-by Step: A Comprehensive Approach to Case Management [Internet]. Homeless Hub. 2021 [cited 2022 Jun 2]. Available from: https://www.homelesshub.ca/resource/step-step-comprehensive-approach-case-management

14. Mueser KT, Bond GR, Drake RE, Resnick SG. Models of Community Care for Severe Mental Illness: A Review of Research on Case Management. Schizophr Bull [Internet]. 1998 Jan 1;24(1):37–74. Available from: https://academic.oup.com/schizophreniabulletin/article-lookup/doi/10.1093/oxfordjournals.schbul.a033314

15. Government of Canada. Backgrounder-Youth Employment Strategy [Internet]. Employment and Social Development. 2022 [cited 2022 Jun 1]. Available from: https://www.canada.ca/en/employment-social-development/news/2017/05/backgrounder_youthemploymentstrategy.html

16. Grace M, Coventry L, Batterham D. The role of interagency collaboration in “joined-up” case management. J Interprof Care [Internet]. 2012 Mar 6;26(2):141–9. Available from: http://www.tandfonline.com/doi/full/10.3109/13561820.2011.637646

17. Roth AJ, Kornblith AB, Batel-Copel L, Peabody E, Scher HI, Holland JC. Rapid screening for psychologic distress in men with prostate carcinoma: a pilot study. Cancer. 1998 May;82(10):1904–8.

18. K. Ownby, PhD, RN, ACHPN, AOCN, ANP-BC K. Use of the Distress Thermometer in Clinical Practice. J Adv Pract Oncol. 2019;10(2):175–9.

19. Sousa H, Oliveira J, Figueiredo D, Ribeiro O. The clinical utility of the Distress Thermometer in non-oncological contexts: A scoping review. J Clin Nurs. 2021;30(15–16):2131–50.

20. Donovan KA, Grassi L, McGinty HL, Jacobsen PB. Validation of the Distress Thermometer worldwide: state of the science. Psychooncology [Internet]. 2014 Mar;23(3):241–50. Available from: https://onlinelibrary.wiley.com/doi/10.1002/pon.3430

21. Fieger P. Measuring student satisfaction from the Student Outcomes Survey. Natl Cent Vocat Educ Res [Internet]. 2012;1–17. Available from: https://www.ncver.edu.au/research-and-statistics/publications/all-publications/measuring-student-satisfaction-from-the-student-outcomes-survey

22. Wiener L, Battles H, Bedoya SZ, Baldwin A, Widemann BC, Pao M. Identifying Symptoms of Distress in Youth Living with Neurofibromatosis Type 1 (NF1). J Genet Couns [Internet]. 2018 Feb 23;27(1):115–23. Available from: http://doi.wiley.com/10.1007/s10897-017-0128-1

23. Donner A, Klar N. Pitfalls of and Controversies in Cluster Randomization Trials. Am J Public Health. 2004;94(3):416–22.

24. Sedaghat AR. Understanding the Minimal Clinically Important Difference (MCID) of Patient-Reported Outcome Measures. Otolaryngol Neck Surg [Internet]. 2019 Oct 4;161(4):551–60. Available from: http://journals.sagepub.com/doi/10.1177/0194599819852604

25. Harris PA, Taylor R, Thielke R, Payne J, Gonzalez N, Conde JG. Research electronic data capture (REDCap)—A metadata-driven methodology and workflow process for providing translational research informatics support. J Biomed Inform [Internet]. 2009 Apr;42(2):377–81. Available from: https://linkinghub.elsevier.com/retrieve/pii/S1532046408001226

26. Harris PA, Taylor R, Minor BL, Elliott V, Fernandez M, O’Neal L, et al. The REDCap consortium: Building an international community of software platform partners. J Biomed Inform [Internet]. 2019 Jul;95:103208. Available from: https://linkinghub.elsevier.com/retrieve/pii/S1532046419301261

27. Ministry of Health & Long-Term Care. Personal Health Information Protection Act [Internet]. 2004 [cited 2020 Dec 8]. Available from: https://www.ontario.ca/laws/statute/04p03

28. Human Resources and Skills Development Canada. Essential Skills Self-Assessment for the Trades [Internet]. Essential Skills and Apprenticeship. 2009 [cited 2022 May 31]. Available from: https://publications.gc.ca/collections/collection_2010/rhdcc-hrsdc/HS18-9-3-2009-eng.pdf

# 34 Budget

| Cost Component | Persons Involved | Total time (hours) | Cost per  hour | Total Cost ($) |
| --- | --- | --- | --- | --- |
| **Study-related products & training** |  |  |  |  |
| Enrollment logs/Participant master files  Randomization module  Electronic case report form  Standard operating procedures  Training manuals | Centre for Education & Research (CER)  CER  Research coordinator | 20  20  20 | 150  100  40.50 | 3 000  2 000  810 |
| Engagement & training | Research coordinator | 40 | 40.50 | 1620 |
| Research personnel business cards | Materials | N/A | N/A | 1000 |
| Research ethics board application | Research coordinator | 10 | 40.50 | 405 |
| Protocol review prior to research ethics board submission | Research manager | N/A | N/A | 700 |
| Research Ethics Board Fee | Research Ethics Board | N/A | N/A | 500 |
| Distress Thermometer Tool  Development  Microsoft Azure hosting  Microsoft Azure technical support | Georgian College  Microsoft Azure  Microsoft Azure | 200  10 years  3 years | N/A  $300/yr  $200/yr | 15 000  3 000  600 |
| Apple iPADs |  | 24 units |  | 24 000 |
| *Subtotal* |  |  |  | **52 635** |
| **Trial Management** |  |  |  |  |
| Informed consent  Enrollment  Randomization | Research coordinator | 800 (2 hr/pt) | 40.50 | 32 400 |
| Survey dissemination/completion | Research coordinator | 400 (1 hr/patient) | 40.50 | 16 200 |
| Case report form completion | Research coordinator | 800 (1 hr/patient) | 40.50 | 32 400 |
| Site/data audit/close out | Research coordinator | 90 (15 hr/6 months) | 40.50 | 3 645 |
| *Subtotal* |  |  |  | **84 645** |
| **Data Analysis** |  |  |  |  |
| Cleaning  Statistical analysis  Data report | CER | 40 | 150 | 6 000 |
| Healthcare utilization data | ICES | N/A | N/A | 15 000 |
| *Subtotal* |  |  |  | **21 000** |
| **Report** |  |  |  |  |
| Publication costs (protocol and final publication) | Journal fees | N/A | N/A | 5 000 |
| *Subtotal* |  |  |  | **5 000** |
| **Incentives** |  |  |  |  |
| Cash incentive | Participants | 200 | $50/participant | **10 000** |
| ***Final Costs*** |  |  |  | ***168285*** |

# 35 Appendix

## 35.1 National Centre for Vocational Education Research Student Outcomes Survey

The responses to all questions are: Strongly disagree; disagree; neither; agree; Strongly agree; N/A

| **Teaching** |
| --- |
| My instructors had a thorough knowledge of the subject content |
| My instructors provided opportunities to ask questions |
| My instructors treated me with respect |
| My instructors understood my learning needs |
| My instructors communicated the subject content effectively |
| My instructors made the subject as interesting as possible |
| **Assessment** |
| I knew how I was going to be assessed |
| The way I was assessed was a fair test of my skills |
| I was assessed at appropriate intervals |
| I received useful feedback on my assessment |
| The assessment was a good test of what I was taught |
| **Generic skills and learning experiences** |
| My training developed my problem-solving skills |
| My training helped me develop my ability to work as a team member |
| My training improved my skills in written communication |
| My training helped me to develop the ability to plan my own work |
| As a result of my training, I feel more confident about tackling unfamiliar problems |
| My training has made me more confident about my ability to learn |
| As a result of my training, I am more positive about achieving my goals |
| My training has helped me think about new opportunities in life |
| **Overall satisfaction with the training** |
| Overall, I was satisfied with the quality of this training |

## 35.2 Foundational skills assessment

Link for questionnaire

<https://publications.gc.ca/collections/collection_2010/rhdcc-hrsdc/HS18-9-3-2009-eng.pdf>

## 35.3 Distress Thermometer

The following are selected screenshots from the tool that will be used in this study (from a hypothetical participant(s)).

Miscellaneous screenshots


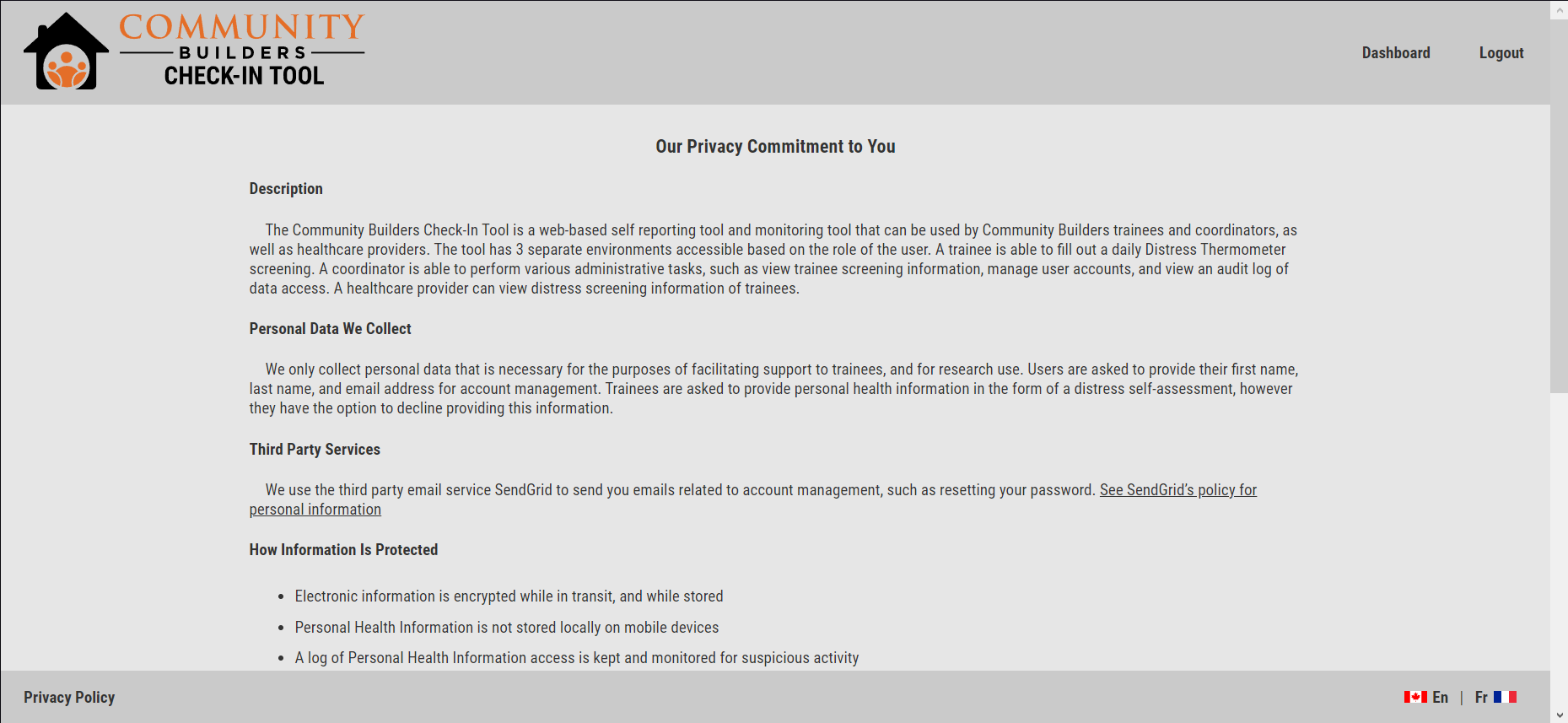


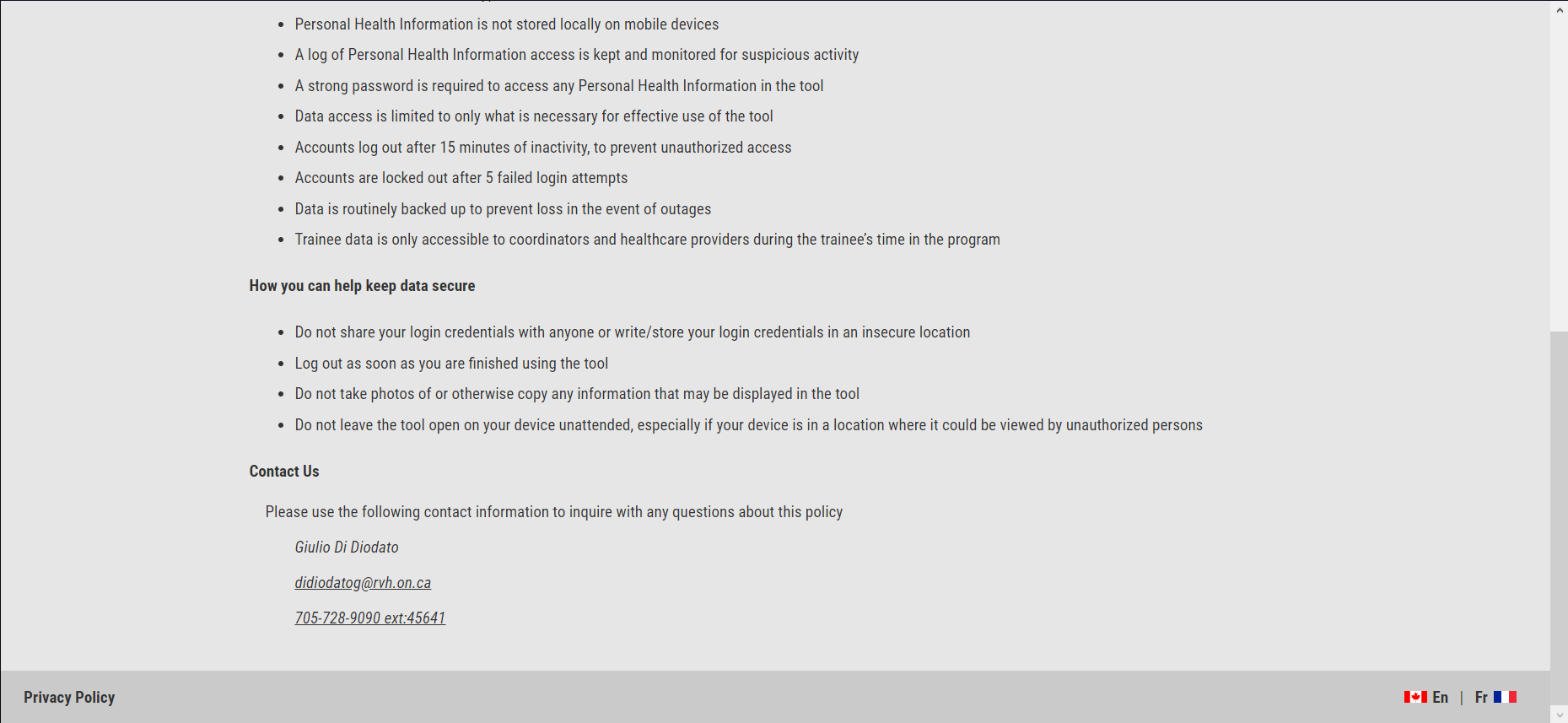


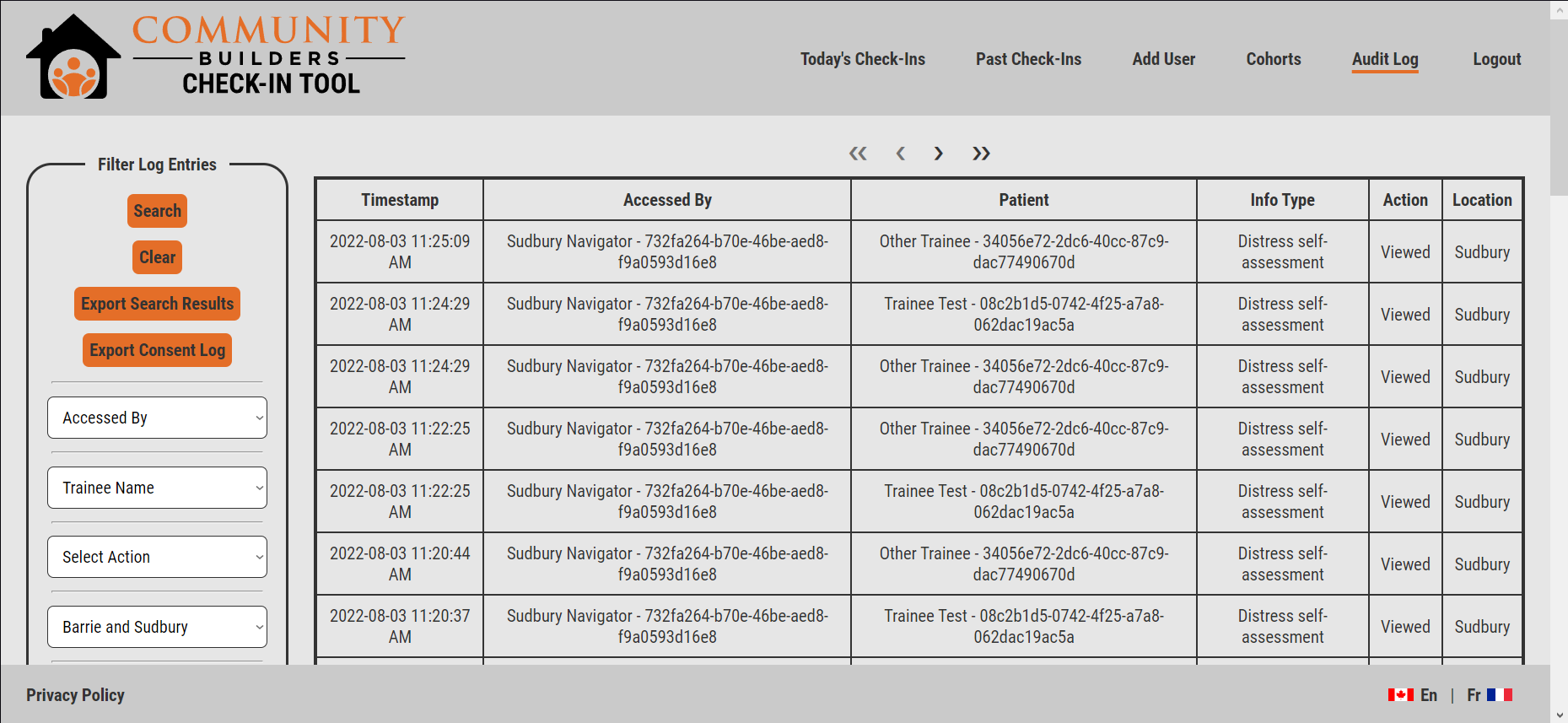


## 35.4 Expressed Consent Presentation
